# Improving Italian general practice training: the role of academia

**DOI:** 10.3399/bjgpopen17X100989

**Published:** 2017-06-14

**Authors:** Luca Cegolon, William C Heymann, John H Lange, Carla Xodo

**Affiliations:** 1 Scientific Directorate, IRCCS Burlo Garofolo, Trieste, Italy; 2 Primary Care Unit, Local Health Unit N.4, Veneto Region, Italy; 3 Florida Department of Health, Sarasota County Heath Department, Sarasota, FL, US; 4 Department of Clinical Sciences, Florida State University, College of Medicine, Sarasota, FL, US; 5 Envirosafe Training and Consultants, Pittsburgh, PA, US; 6 FISPPA Department, Padua University, Padua, Italy

**Keywords:** general practice training, Italy, European Union, family medicine, research, post graduate specialist training, academic primary care

## Background

The modern Italian national health service is a Beveridgean system centred around GPs and funded by central taxation.^1,2^ In order to become eligible to work as a GP, Italian doctors need to undertake a 3-year postgraduate training in general practice.^3–6^ The latter training — governed nationally by the Italian Ministry of Health and locally by the various Italian regions — is organised by the primary care establishment.^3–6^ By contrast, all other postgraduate medical specialties in Italy are organised by universities and are governed by the Ministry of Education, University and Research (MIUR).

On successful conclusion of the above GP training, Italian doctors are awarded a certificate of completion of training which is then mutually recognised in all member states of the European Union (EU).^3–5^ Italian doctors in postgraduate university specialty training (that is, non-GP trainees) had not benefited from any financial support from the government during their training until 1991. From 1992 until 2000 a tax-free monthly studentship of about 960 000 Italian Lira was introduced to support non-GP trainees; the latter bursary then became approximately €960 from 2000 to 2006 (after the introduction the Euro currency to Italy). Proper working contracts with an annual gross salary of €22 700 (a variable monthly amount of €1600–1700 after taxes) for this category of specialist trainees started in 2007. As a result, the Italian Supreme Court recently ordered the Italian government to pay compensation to Italian doctors training for medical specialities governed by the university authority from 1 January 1983 onward, in compliance with the Directives of the Council of the EU regarding stipends of doctors during postgraduate specialist training. The latter directives were accompanied by related decisions of regional tribunals. Unfortunately, these decisions concerned only doctors in training in secondary care specialties (that is, non-GP trainees), while Italian GP trainees currently still lack working contracts for their postgraduate clinical training in family medicine and are dependent on an annual studentship of €11 600 (about €800 per month after taxes), which was introduced back in 1992, similarly to medical specialties under university governance.^3,7^

## Discussion

In addition to GP trainees being paid less than their hospital trainee counterparts and a lack of a working contract, they are prohibited from any other out-of-hours self-employed remunerated medical activity during their 3-year postgraduate training in family medicine. Breaching this regulation implies criminal charges of 'fraud', the expulsion from the GP training programme, and the obligation to pay back all salaries earned up to the date of expulsion.^6^

A change in the law in 2010 was positively innovative, as it combined postgraduate university specialty medical training (that is, non-GP training) with concurrent doctoral studies, thus allowing completion of training in a shorter period of time. In several countries, such as the UK, US, and Australia, the importance attributed to integrated training has resulted in joint MBBS/PhD programmes which confer a dual doctorate of medicine and philosophy, thus enhancing professional life-years. Unfortunately, these changes in the Italian medical training system are not applicable to doctoral studies in family medicine. GP trainees are also banned from registering for any PhD programmes during the 3-year training in primary care. Breaching this regulation is another criminal offense, which results in expulsion from the postgraduate training scheme in family medicine and the obligation to pay back all salaries earned up to the date of expulsion.^6^

This could invite legal arguments against unfair treatment of Italian GP trainees compared with their colleagues training in a university medical specialty. The reasons for this ongoing discriminatory situation, which is detrimental to Italian GP trainees, are still largely unexplained, but there is a widespread opinion that this is the result of 'politics'. In the contractual negotiations with the Italian government, the powerful action of the GP unions seems more focused on protecting the independence of the GP category from university control and the professional privileges of senior GPs.^8^

Another more serious criticism is the quality of education delivered during this 3-year postgraduate training scheme in family medicine, in terms of seminars and practical clinical education. As a result, the professional standing of GPs within the Italian medical community is largely perceived as downgraded.^9–11^ Furthermore, despite some reports, no formal undergraduate primary care curriculum is provided in Italian medical schools.^12,13^

General practice training has been mandatory in all member states of the EU since 1986. As further confirmed in Directive 93/16/EEC3, all member states had to fully comply with this legislation by 1 January 1995. The EU Directive also defined the minimum acceptable length of GP training and requires reciprocal recognition of GP diplomas across EU member states. Postgraduate GP training in Italy was introduced back in 1988,^7^ as a preferential (non-essential) curricular element to practice as a GP, and it was initially structured as a 2-year scheme, becoming a 3-year programme from 2003 onwards. The Legislative Decree 256/1991 subsequently ruled that the GP training was a basic requirement (that is, no longer optional) from January 1995 onward for Italian doctors wishing to practice as GPs. ^4–7^ Nevertheless Italian doctors obtaining their MBBS by December 1994 are exempt from this requirement and eligible to practice as a GP under previously acquired rights, without further training and qualifications in addition to the MBBS and the licence to practice medicine within the country. Within each Italian region the GP training is managed by a consortium of local GPs. With regard to professional qualifications or the ability to teach, selective standards for tutors involved in seminars and teaching activities are not considered in the recruitment criteria.^8–11^ Since formal academic training in family medicine is missing and is not a requirement, primary care clinical tutors of Italian general practice schools are usually experienced GPs, but without academic experience, who have not the opportunity to develop formal educational and teaching skills, and are not actively involved in research and publishing. During their clinical training, Italian GP trainees also rotate through health districts to learn organisational aspects of the primary care service and through hospital wards to learn practical elements of secondary care.^4–5^ These trainee doctors attend hospital wards reportedly under unsatisfactory supervision,^9^ which is carried out by a tutor from each secondary care department generally in bureaucratic fashion.^8–11^ There is in fact no obligation for an in-depth evaluation of the GP trainee at the end of each rotation in hospital wards. Rather than a monocratic appraisal by a single tutor, the acquisition of new expected competencies by the GP trainee should be better conducted in presence of a neutral coutersignatory or by a small panel, where the tutor would be supported by external figures or experts to verify and supervise the appraisal procedure following an evidence-based approach.^8–11^

These various aspects of the GP training may be further amplified with reference to research. Since there are no primary care departments in Italian medical schools, there are no opportunities to pursue a PhD in family medicine in the country. Research in primary care is conducted only by isolated centres, such as the Mario Negri Institute for Pharmacological Research in Milan. There is no provision to develop a GP network with an accessible national database for population-based studies, such as the UK Clinical Practice Research Datalink or the UK Biobank.

Nonetheless, research in primary care is vital as it directly supports disease prevention and promotes evidence-based health care and health promotion. Patients with chronic conditions often have multiple comorbidities requiring attention and are therefore more suitably managed at the primary care level rather than being referred to multiple medical specialists. Family doctors commonly manage a large number of patients with long-term conditions and often facilitate the entry of patients into the national health service in Italy, while providing continuity of care.^14,15^ As a result, GPs have become the main source not only of primary care, but also of epidemiological data, and can contribute to population-based studies.^16^ Research in primary care might also serve as a good motivator for ambulatory practice, although financial incentives are essential instruments to motivate GPs and attain quality health outcomes.^17^

Unlike the Italian situation, in most EU countries academic general practice pathways are now available and considerable progress has been made in this field over the past 20 years. Northern European countries, including the Scandinavian countries, the Netherlands, and the UK are leading the way. Almost all post-communist Central and Eastern European countries have set up primary care departments and included family medicine in the undergraduate medical curriculum.^18^ Most Southern European countries, namely Portugal, Greece, France, Slovenia, Croatia, and Malta have also developed academic GP training curricula.^8,18–20^ In 2016 the Spanish Academy of Family Medicine was also established.^21^ Italy, therefore, is currently the only Southern member of the EU still without academic general practice. Setting up an academic pathway for Italian GP training is therefore an essential turning point to keep up with the best practice in the rest of Europe.

## Conclusion

The main issues that need to be addressed regarding Italian general practice training are summarised as follows:

- eliminating the disparities between GP trainees and university medical specialty trainees;
- establishing primary care research;
- creating better qualified GPs; and
- attracting the best medical students and strengthening the reputation of family medicine.

Are there viable solutions? Reasonable actions that may be considered include:^8,22,23^

- creating a single unified national medical council;
- establishing stronger regulation of family medicine at the national level;
- introducing measures for external quality control of GP training;
- adapting the GP training provided to that in other EU countries;
- introducing a national undergraduate medical curriculum for family medicine;
- developing academic primary care departments with career positions; and
- introducing a research doctorate with a thesis in the field of primary care as a basic requirement for a GP professorship.
